# An integrated behavioral treatment for improving nocturia and insomnia symptoms in older adults (MINT): study protocol for a multi-site randomized clinical trial

**DOI:** 10.1186/s13063-026-09464-2

**Published:** 2026-02-16

**Authors:** Constance H. Fung, Courtney J. Bolstad, Alison Huang, Alayne Markland, Jing Cheng, Cathy Alessi, Theodore M. Johnson, Donald L. Bliwise, Jennifer L. Martin, Kathryn L. Burgio, Michael Schembri, Erin Der-McLeod, Taressa Sergent, Camille P. Vaughan

**Affiliations:** 1 Geriatric Research, Education and Clinical Center, VA Greater Los Angeles Healthcare System, Los Angeles, California USA; 2 Department of Medicine, University of California, Los Angeles, CA USA; 3 Birmingham/Atlanta Geriatric Research, Education and Clinical Center, Birmingham VA Health Care System, Birmingham, Alabama USA; 4 Division of Gerontology, Geriatrics, and Palliative Care, University of Alabama at Birmingham, Birmingham, AL USA; 5 Departments of Medicine, Urology, and Epidemiology and Biostatistics, University of California San Francisco, San Francisco, California USA; 6 Division of Geriatrics, University of Utah School of Medicine, Salt Lake City, Utah USA; 7 Geriatric Research, Education, and Clinical Center, Salt Lake City VA Healthcare System, Salt Lake City, UT USA; 8 Division of Oral Epidemiology and Dental Public Health, University of California San Francisco, San Francisco, California USA; 9 Department of Family and Preventive Medicine, Emory University, Atlanta, GA USA; 10 Sleep Program, Emory University School of Medicine, Atlanta, GA USA; 11 Department of Medicine, University of California San Francisco, San Francisco, CA USA; 12 Birmingham/Atlanta Geriatric Research, Education, and Clinical Center, Atlanta VA Healthcare System, Atlanta, Georgia, USA; 13 Division of Geriatrics & Gerontology, Department of Medicine, Emory University School of Medicine, Atlanta, GA USA

**Keywords:** Nocturia, Insomnia, Behavioral intervention, Older adult

## Abstract

**Background:**

Nocturia (i.e., waking to void during the primary sleep period) of two or more times per night affects nearly one-third of older adults and can have a severe impact on sleep, contributing to insomnia symptoms. Current treatment approaches for nocturia often overlook non-lower urinary tract factors that may contribute to nighttime awakenings. Nocturia management, for example, may benefit from more effective integration of cognitive behavioral therapy for insomnia (CBT-I) principles that address other factors underlying insomnia symptoms, and early evidence suggests it also reduces nocturia and the bother it causes. Because nocturia treatment crosses specialties, coordinated delivery of urological and sleep therapies is a treatment barrier. The overall purpose of this trial is to determine whether a promising coordinated, integrated behavioral, non-pharmacological, non-surgical treatment that simultaneously addresses both the urological and insomnia factors contributing to nocturia is efficacious for improving nocturia, sleep, and daytime function.

**Methods:**

This multicenter parallel-group randomized, efficacy trial compares a 5-week integrated behavioral treatment program delivered by a single interventionist (psychologist, nurse practitioner, or physician assistant) to a health education control program in adults aged 60 years or older (proposed *n* = 192) recruited from sites in Atlanta and Los Angeles, who report typically getting up to urinate two or more times per night (International Consultation on Incontinence Questionnaire-Overactive bladder [ICIQ-OAB] nocturia item) and insomnia symptoms (Insomnia Severity Index > 7). The integrated program includes components of CBT-I and pelvic floor muscle exercise-based behavioral therapy for nocturia. The primary outcome is ICIQ-OAB-measured nocturia frequency 4 months after randomization. Secondary outcomes are sleep diary-measured wake after sleep onset (mean minutes) and Insomnia Severity Index total score.

**Discussion:**

The interdisciplinary trial team has developed a program aimed at improving nocturia symptoms and overall sleep of older adults in an efficient and safe manner. The integrated behavioral program has the potential to address nocturia, which is a challenging symptom because it has many etiologies that cross multiple specialties. Findings will provide rigorous evidence of the efficacy of the integrated behavioral treatment program to reduce nocturia frequency as well as sleep disturbance in older adults.

**Trial registration:**

Clinicaltrials.gov NCT06110091, registered 10/25/2023.

## Administrative information

Note: the numbers in curly brackets in this protocol refer to SPIRIT checklist item numbers. The order of the items has been modified to group similar items (see http://www.equator-network.org/reporting-guidelines/spirit-2013-statement-defining-standard-protocol-items-for-clinical-trials/).

**Table:** 

Title {1}	An integrated behavioral treatment for improving nocturia and insomnia symptoms in older adults (MINT): study protocol for a multi-site randomized clinical trial
Trial registration {2a and 2b}.	clinicaltrials.gov NCT06110091, registered 10/25/2023
Protocol version {3}	March 10, 2025 (v6)
Funding {4}	National Institute on Aging (NIA) R01AG083477; in-kind support from Department of Veterans Affairs
Author details {5a}	Constance H. Fung, MD, MSHS, Geriatric Research, Education and Clinical Center, VA Greater Los Angeles Healthcare System; Department of Medicine, University of California, Los Angeles, Los Angeles, California Courtney J. Bolstad, PhD, Birmingham/Atlanta Geriatric Research, Education and Clinical Center, Birmingham VA Health Care System; Division of Gerontology, Geriatrics, and Palliative Care, University of Alabama at Birmingham, Birmingham, Alabama Alison Huang, MD, MAS, MPhil, Departments of Medicine, Urology, and Epidemiology and Biostatistics, University of California San Francisco, San Francisco, California Alayne Markland, DO, MSc, Division of Geriatrics at the University of Utah School of Medicine; Salt Lake City Geriatric Research, Education, and Clinical Center, Salt Lake City VA Healthcare System, Salt Lake City, Utah Jing Cheng, PhD, Division of Oral Epidemiology and Dental Public Health, University of California San Francisco, San Francisco, California Cathy Alessi, MD, Geriatric Research, Education and Clinical Center, VA Greater Los Angeles Healthcare System; Department of Medicine, University of California, Los Angeles, Los Angeles, California Theodore M. Johnson II, MD, MPH, Department of Family and Preventive Medicine, Emory University, Atlanta, Georgia Donald L. Bliwise, PhD, Sleep Program, Emory University School of Medicine, Atlanta, Georgia Jennifer L. Martin, PhD, Geriatric Research, Education and Clinical Center, VA Greater Los Angeles Healthcare System; Department of Medicine, University of California, Los Angeles, Los Angeles, California Kathryn L. Burgio, PhD, Division of Gerontology, Geriatrics, and Palliative Care, University of Alabama at Birmingham; Birmingham/Atlanta Geriatric Research, Education and Clinical Center, Birmingham, Alabama Michael Schembri, BS, Departments of Medicine, University of California San Francisco, San Francisco, California Erin Der-McLeod, MSW, Geriatric Research, Education and Clinical Center, VA Greater Los Angeles Healthcare System, Los Angeles, California Taressa Sergent, BA, Birmingham/Atlanta Geriatric Research, Education, and Clinical Center, Atlanta VA Healthcare System; Decatur, Georgia Camille P. Vaughan, MD, MS, Birmingham/Atlanta Geriatric Research, Education, and Clinical Center, Atlanta VA Healthcare System; Division of Geriatrics & Gerontology, Department of Medicine, Emory University School of Medicine, Atlanta, Georgia
Name and contact information for the trial sponsor {5b}	Atlanta VA Medical Center, 1670 Clairmont Rd, Decatur, GA 30033, (404) 321–6111
Role of sponsor {5c}	Several of the measures included in the study follow the requirements outlined by the National Institute on Aging (NIA)’s Clinical Research Operations & Management System (CROMS). The NIA is notified of severe adverse events and has a role in determining whether the trial may be continued. However, the funder does not have any role in other aspects of study design and collection, analysis, interpretation of data, or in writing the manuscript. This material is the result of work supported with resources and the use of facilities at the Atlanta Department of Veterans Affairs (VA) Health Care System, the Greater Los Angeles VA Health Care System. The content is solely the responsibility of the authors and does not necessarily represent the official views of the VA.

## Introduction

### Background and rationale {6a}

Nocturia is a common contributor to poor health outcomes in older adults. Nearly one-third of older adults experience two or more nightly nocturia episodes (i.e., waking to void during primary sleep period) [1, 2]. Despite the usual attribution of the symptom to benign prostate enlargement in men, the prevalence of nocturia is nearly similar among older men and women, although nocturia prevalence increases more dramatically as men age compared to women [3–5]. Nocturia is reported as one of the most bothersome lower urinary tract symptoms [6, 7]. Epidemiological studies indicate that a frequency of two or more episodes of nocturia per night, in particular, is associated with significant “bother” (as measured by its effect on health-related quality of life) and is a clinically meaningful threshold [7, 8]. Nocturia is associated with multiple negative health outcomes, including more incident falls and depression [5, 9–17].

Nocturia, like geriatric syndromes, may be conceptualized as the result of accumulated impairments in multiple systems that render individuals with these conditions vulnerable to situational challenges [18]. The risk factors include biological (e.g., genetic) [19–21], contributing, and perpetuating factors. Contributing factors include those that increase urine production (either over the 24-h period or at nighttime), bladder storage problems (e.g., obstruction, infection), mental health (e.g., depression), medical conditions (e.g., diabetes, obesity) [22], and pain that disrupts sleep. Perpetuating factors include conditioned voiding responses and patient beliefs about fluid intake that may contribute to nocturia. In the proposed model, factors that are well-known contributors to insomnia symptoms, such as excessive time in bed [23], also have a role in perpetuating nocturia and its bother. Several reports suggest that older men and women with nocturia are more likely to find nocturia to be clinically bothersome if it is complicated by poor sleep quality [9, 24]. Studies indicate that nocturia is an independent predictor of insomnia [25], with greater number of trips to the toilet predicting worse patient-reported measures of insomnia (e.g., difficulty initiating sleep and returning to sleep after awakening) as well as objective measures of insomnia (e.g., longer wake bouts, worse sleep efficiency) [24, 26].

Pharmacological treatments for nocturia have modest effects and can be problematic in older adults. These treatments for nocturia (e.g., bladder relaxants, alpha blockers, desmopressin) have either poor (e.g., bladder relaxants, dihydrotestosterone blockers) or modest effects (e.g., alpha blockers) on reducing nocturia frequency [27, 28]. Moreover, medication side effects are common (e.g., desmopressin [arginine vasopressin analog]) [29] and may not be able to be used in patients who already have accumulated impairments in multiple systems, as is common among older adults [30]. Some bladder relaxants, which are often anticholinergic drugs, frequently cause dry mouth and constipation and can lead to delirium or worsened cognition. Some alpha blockers lower blood pressure and increase the risk of orthostatic hypotension, which is a risk factor for dizziness and falls. Desmopressin is associated with significant risk of serious hyponatremia with a black box warning, particularly among older adults [31]. Surgical treatments such as transurethral resection of the prostate may have limited effect on nocturia frequency [32].

Current treatment strategies (i.e., behavioral and drug approaches) for nocturia typically focus exclusively on the bladder or pelvis [33–35], and although these treatments improve nocturia frequency, a major gap is that individuals often continue to wake at night to void even after they have been treated [36, 37]. These findings suggest that additional mechanisms that contribute to nocturia need to be targeted. Non-urinary tract perpetuating factors that contribute to insomnia are viable targets for treatment using components of cognitive behavioral therapy for insomnia (CBT-I) such as sleep restriction, stimulus control, cognitive restructuring, and relaxation training [38, 39]. Consensus group and professional society guidelines recommend assessing for sleep disorders, including insomnia, among patients with nocturia [23, 40]. Recent studies have also focused on whether treatments targeting sleep disturbance result in improved nocturia symptoms. In our work, we found that nocturia frequency independently contributes to poor sleep quality, increased wakefulness at night, and longer time-in-bed, above and beyond other known demographic variables, medical conditions, and depression [41]. In another study, members of our team found that behavioral therapy directed at nocturia symptoms in older adults resulted in modest improvements on insomnia measures (i.e., time to initiate sleep, time to return to sleep after awakening) [36]. Further work has also found that brief behavioral treatment for insomnia reduces the number of self-reported nocturnal voids recorded in sleep diaries compared to an information control in older adults with chronic insomnia (effect size 0.82) [42].

The proposed bidirectional relationship between nocturia and factors that cause insomnia symptoms [43] (and those that may have the potential to cause more disruptive or problematic nocturia) led to our development of a multicomponent, non-pharmacological, non-surgical treatment strategy targeting the perpetuating factors of nocturia and insomnia. In an earlier study, we established that a multicomponent behavioral and lifestyle intervention that addressed sleep disruption in the context of nocturia, incorporating sleep hygiene with nocturia treatments (e.g., pelvic floor muscle exercise-based urge suppression at night), was well-tolerated and feasible [37]. Moreover, behavioral therapy reduced nocturia frequency equal to or at least as much as drug therapy [36, 37, 42, 44]. This evidence of improvement in nocturia frequency was tempered by small improvement in sleep outcomes, including a non-significant change in sleep efficiency and wakefulness after sleep onset [37]. The results suggested a need to add more potent behavioral sleep components (e.g., sleep restriction) to improve treatment of nocturia in older adults.

This work led to the current randomized trial comparing an integrated, multicomponent behavioral intervention to a health education control program in adults aged ≥ 60 with concomitant nocturia and insomnia symptoms to provide rigorous evidence of the efficacy of the integrated program [38]. Informed by our recent trials and prior feasibility study (IRB#16160003), we are recruiting participants using a multi-pronged process designed to limit participant burden while yielding high levels of participation. The 5-week treatment delivered by a single interventionist, which involves an integrated nocturia and insomnia intervention (includes education, lifestyle habits management, cognitive, relaxation, and behavioral conditioning for nocturia and insomnia), addresses the bidirectional relationship between these important and common symptoms.

### Objectives {7}

#### Specific Aim 1

Determine the efficacy of the integrated nocturia and insomnia behavioral intervention program for improving nocturia frequency over 4 months in older adults with nocturia and insomnia symptoms.

##### Hypothesis 1

Compared to a control arm of health education, participants assigned to the integrated intervention program will demonstrate a greater reduction in mean nocturia frequency over 4 months.

#### Specific Aim 2

Assess the efficacy of the integrated nocturia and insomnia behavioral intervention for reducing sleep disturbance over 4 months in older adults with nocturia and insomnia symptoms.

##### Hypothesis 2

Compared to a health education control arm, participants assigned to the integrated intervention program will have greater reduction (i.e., improvement) in self-reported wake after sleep onset and Insomnia Severity Index over the 4-month follow-up period.

#### Specific Aim 3

Examine the effects of the integrated nocturia and insomnia behavioral intervention on quality of life (daytime function) related to nocturia and sleep quality over 4 months in older adults with nocturia and insomnia symptoms.

##### Hypothesis 3

Participants randomized to the integrated intervention program will report more improvement in Nocturia-Quality of Life scores and Pittsburgh Sleep Quality Index Global Score (lower scores) from baseline to post-treatment and 4-month follow-up compared to control.

### Trial design {8}

Randomized parallel group superiority trial (1:1 allocation).

## Methods: participants, interventions and outcomes

### Study setting {9}

Participant recruitment is based at two sites within the United States: The Department of Veterans Affairs (VA) Greater Los Angeles and the Atlanta VA, within the Geriatric Research, Education and Clinical Centers and respective outpatient clinics at each site. Additionally, patients are recruited from outpatient clinics at two universities affiliated with these VA centers, the University of California Los Angeles (UCLA) Health System and Emory University. Data coordination activities are based at the University of California, San Francisco (UCSF). Additional quality control for intervention (e.g., fidelity ratings) is conducted by co-investigators at the Birmingham VA and the University of Utah/Salt Lake City VA. This list of study sites is comprehensive and is also available from the corresponding author.

### Eligibility criteria {10}

Participant eligibility criteria include the following:

Inclusion criteria:

- Age ≥ 60 years
- Average of ≥ 2 episodes per night of nocturia on the International Consultation on Incontinence Questionnaire-Overactive Bladder Module (ICIQ-OAB) questionnaire
- Insomnia Severity Index > 7
- Able to attend weekly in-person or virtual study visits

Exclusion criteria:

- Presence of bipolar disorder
- Significant cognitive impairment as measured by a score < 20 on the Mini-Mental State Examination
- Sleep disturbance better explained by another sleep disorder such as restless legs syndrome, narcolepsy, insufficient sleep syndrome, or circadian rhythm sleep-wake disorders
- Untreated sleep-disordered breathing (respiratory event index ≥ 15 plus Epworth Sleepiness Scale > 10, or respiratory event index ≥ 30. Note that participants with treated sleep-disordered breathing are *not* excluded)
- Current urinary tract infection or hematuria (based on screening urinalysis testing)
- Unstable doses or recent changes (past month) in medications affecting lower urinary tract symptoms
- New or recently discontinued insomnia medication within the past month
- Previous or current intensive behavioral therapy for insomnia or urinary symptoms
- Unstable health conditions expected to result in death or hospitalization within 3 months
- Unstable medical conditions that could contribute to nocturia or insomnia such as poorly controlled heart failure as evidenced on physical examination, poorly controlled diabetes mellitus with either hemoglobin A1c of ≥ 9.0, or chronic kidney disease (stage 4 or 5) or a potential to initiate dialysis in 3 months
- Unstable psychiatric conditions (e.g., psychosis, active alcohol/substance abuse based on history and medical records)
- Unstable housing situation
- Evidence of significant urinary retention (e.g., as measured by a residual bladder volume of ≥ 200 mL by bladder ultrasound or catheterization with 15 min of voiding)
- Genitourinary cancer undergoing active treatment
- Pelvic or colon surgery within 6 months of enrollment or onabotulinum toxin therapy for urinary symptoms within 6 months of enrollment

Interventionist eligibility criteria: The intervention protocols were designed for delivery by a clinician who may be an advanced practice practitioner (e.g., nurse practitioner or physician assistant who delivers behavioral treatment in a urology clinic) or a clinical psychologist (e.g., behavioral sleep specialist who delivers cognitive behavioral therapy for insomnia in a sleep center) and who is fully trained in the intervention protocols by the study clinician scientists. Including clinicians with diverse training and background will facilitate future dissemination, since our goal is to develop a cross-disciplinary intervention program.

### Who will take informed consent? {26a}

Research coordinators working under the supervision of clinician Site PIs, using an institutional review board (IRB)-approved process, meet (either in-person or through a video visit) with prospective participants who have undergone preliminary screening either by telephone or in-person. When a prospective participant attends the consent visit, a research staff member discusses the study details with the prospective participant and assesses their capacity to sign the informed consent forms by asking the prospective participant to recount the main purpose of the trial, risks and benefits of participation, and options if the individual no longer wishes to participate. They may sign the informed consent forms (either through e-consent platform approved by the institutions or with a wet signature). Proxy is not pursued for individuals who do not have the capacity to provide informed consent.

### Additional consent provisions for collection and use of participant data and biological specimens {26b}

No biological specimens are collected for analysis. Home sleep apnea test results that suggest sleep apnea diagnosis may be shared with the participant’s clinical team, if the participant gives the research team permission to share the results.

## Interventions

### Explanation for the choice of comparators {6b}

The integrated behavioral intervention is compared to a healthy aging education control program as an attention control condition, to address the goal of the project to establish the initial efficacy of the integrated behavioral intervention. The two programs were selected to be comparable in number of sessions, to be behavioral in nature (non-pharmacological, non-surgical), to be provided through a video visit or in-person visit over 5 visits in addition to up to 2 telephone check-in calls, to be delivered entirely (all sessions) by a single interventionist, and to have a structured format (slide presentation, binder with handouts, and “homework” between sessions).

### Intervention description {11a}

#### Integrated insomnia and nocturia treatment program

The integrated behavioral treatment program was designed by an interdisciplinary team of nocturia and insomnia experts to guide and support older adults in examining their current sleep and bladder habits, practicing strategies to improve their sleep and reduce nighttime trips to the bathroom, and discovering ways to use these strategies long term [38]. The program is designed to deliver core content across 5 treatment sessions (approximately 60 min per session) with an interventionist over 5 weeks as well as up to 2 brief telephone check-ins after 5 weeks to support participants in continuing to practice intervention techniques on their own.

Session 1 provides an introduction to treatment and education on the science of sleep and nighttime bladder control. Participant goals for Session 1 include understanding the rationale of the program and expectations for session and inter-session activities as well as gaining knowledge about their sleep and bladder control. Session 1 activities include discussing the science of sleep and urination and the bidirectional relation between the bladder and sleep (the focus on the bidirectional relationship is an innovative aspect of the integrated program), a review of the participant’s sleep/bladder diary, and setting a new sleep schedule (e.g., sleep restriction).

Session 2 focuses on healthy steps for sleep and bladder control. At the end of Session 2 participants should be able to use pelvic floor muscle exercises and urge suppression, including at night; identify habits, conditions, and medication that worsen sleep and nocturia as well as use strategies to modify these factors to improve sleep and nocturia; understand stimulus control and winddown routines; and plan ahead for behavior following nighttime awakenings. Session 2 activities include discussion of bedtime and nighttime activities that affect sleep and the bladder as well as making changes to the participant’s sleep schedule. Session 2 includes innovative content linking daytime exercises and urges suppression techniques to nighttime sleep.

Session 3 targets naps and stress and expands strategies related to the bladder. Participant goals for Session 3 include understanding the impact of napping on nighttime sleep and homeostatic control; understanding stress and sleep as well as tactics for reducing physiological and cognitive arousal; and further developing skills for relaxing the bladder and decreasing the sensation to urinate. Activities in Session 3 include checking in/troubleshooting; discussion of how napping affects nighttime sleep; introduction of a tool for participant to identify their level of stress across the day; stress management techniques; and review of pelvic floor muscle exercises and urge suppression techniques. Use of the Freeze and Squeeze urge suppression strategy in response to urges to void at night is an example of innovation included in Session 3.

Session 4 targets thoughts and behaviors related to sleep and the bladder. By the end of the session, participants should be able to identify ways to manage counterproductive thoughts and behaviors related to sleep and nighttime voiding as well as develop a better understanding of prior session content (Freeze and Squeeze, stimulus control, sleep restriction). Session 4 activities include education on thoughts, behavior, and emotions; a review of pelvic floor muscle exercises and urge suppression technique progress; a review of Session 3 materials; checking in/trouble-shooting stress management; review of the participant’s sleep/bladder diary; and creating a weekly plan for sleep and bladder. Innovation in Session 4 includes highlighting connection between thoughts, sleep, and bladder (mind over bladder) and healthier ways to manage these three components.

Session 5 focuses on participants’ progress and preparation for the future. Goals of Session 5 include highlighting progress and identifying participant’s information gaps; discussing the participant’s plan for future scenarios and how to manage symptoms, and discussion of next steps. Activities in Session 5 include discussion of what the participant has learned and how their sleep and nocturia are now; discussion of what to do when the participant has sleep or bladder issues in the future; review of the participant’s accomplishments; and discussion of the next appointments.

Brief telephone check-ins occur up to twice after the participant has completed the integrated program. The goal of these calls is to check in on the participant’s current sleep schedule (time in bed window), stimulus control, Freeze and Squeeze activity, and management of thoughts about sleep and voiding. Activities also include a selective review of prior material based on current complaints.

#### Healthy aging education control arm

Participants attend visits with the interventionist to control for time and attention, which enhances the scientific rigor of our trial. The interventionist provides a health education curriculum. The health education modules focus on brain health and include topics such physical activity, social engagement, sleep topics (e.g., changes in sleep that occur with aging, effects of poor sleep on health), medications/medical conditions, vision and hearing impairment. Brief telephone check-ins are comprised of review of health topics discussed during the sessions. Goals (e.g., physical activity, track medications daily) are established at the visits, and progress towards these goals is monitored in a workbook that mirrors the sleep/bladder diary of the integrated program but does not include items from the sleep/bladder diary because it is considered a self-monitoring intervention. Brief telephone check-ins (up to two) are conducted to discuss progress on goals previously established during the 5 content sessions.

### Criteria for discontinuing or modifying allocated interventions {11b}

#### Criteria for modifying allocated interventions

The amount of sleep restriction, which is one of the components of the integrated behavioral intervention, is modified if the participant has high levels of excessive daytime sleepiness or excessive daytime sleepiness that is negatively impacting their daytime function. Stimulus control recommendations may be modified for individuals who cannot independently transfer out of bed at night. If sleepiness levels increase during the intervention, additional modifications will be made to the sleep schedule to extend time in bed. For the healthy aging education program, participants with concerns about engaging in more physical activity (e.g., due to health conditions) are not advised to increase their physical activity.

#### Criteria for discontinuing allocated interventions

Either program may be discontinued if a participant requests discontinuation. The program may be discontinued by the investigators if a participant refuses to follow safety precautions (e.g., regarding sleepiness).

### Strategies to improve adherence to interventions {11c}

To improve adherence to the intervention protocols, participants in both study arms are provided with a binder with program material, including forms for participants to encourage more active participation and adherence to the intervention protocols.

To monitor adherence to the integrated behavioral program, participants complete a daily sleep/bladder diary, which they share with the interventionist. The diary enables the team to monitor adherence to critical elements such as the actual time-in-bed window, which may be compared to the prescribed time-in-bed window documented in the interventionist’s session notes. A survey about adherence and self-efficacy for performing exercises taught in the integrated behavioral program is also assessed at the final session.

### Relevant concomitant care permitted or prohibited during the trial {11d}

Participants are advised to refrain from beginning new bladder or sleep medications during the trial or to begin cognitive behavioral therapy for insomnia or behavioral treatment for nocturia through non-research providers. This information is assessed at the follow-up visits. Continued use of medications that may affect lower urinary tract symptoms or sleep, including diuretics and sedatives, or medications with sedating effects are permitted.

### Provisions for post-trial care {30}

Participants are being actively recruited from clinical practices and will have access to routine care. Participants who are injured as a result of taking part in the trial will receive the necessary medical treatment at no cost from the Department of Veterans Affairs unless the injury is due to nonadherence by the study participant with study procedures.

### Outcomes {12}

#### Primary outcome: questionnaire-assessed nocturia frequency

Specific measurement variable: Nocturia frequency is assessed as a primary outcome using an item from the ICIQ-OAB, which is a 4-item instrument that measures nocturia, urgency, frequency, urinary incontinence symptoms, and bother [45, 46]. It is derived from the International Continence Society (ICS) male and Bristol Female Lower Urinary Tract Symptoms questionnaires. It has Grade A validation due to its psychometric testing and published data sets demonstrating validity, reliability, and responsiveness [47].

- Analysis metric: nocturia frequency (4 months post-randomization)
- Method of aggregation: mean
- Time point: 4 months post-randomization

#### Secondary outcome: diary-assessed nocturia frequency

Specific measurement variable: Nocturia frequency is also assessed with the 7-day Sleep/Bladder Assessment Diary for diaries with ≥ 5 days of data. The nocturia diary items include participant-reported voids. Voids are determined to occur during the sleep period based on participant-recorded sleep and wake times.

- Analysis metric: nocturia frequency (4 months post-randomization)
- Method of aggregation: mean
- Time point: 4 months post-randomization

#### Secondary outcome: diary-assessed maintenance insomnia

Specific measurement variable: Wake after sleep onset is derived from the 7-day Sleep/Bladder Assessment Diary for diaries with ≥ 5 days of data. The sleep diary items are adapted from American Academy of Sleep Medicine Consensus Sleep Diary [48] for daily sleep schedule, habits, and medication use. A difference of 20 min between treatment and control is considered the clinical significance threshold for studies of chronic insomnia disorder [49].

- Analysis metric: wake after sleep onset (4 months post-randomization)
- Method of aggregation: mean
- Time point: 4 months post-randomization

#### Secondary outcome: insomnia severity

Specific measurement variable: Insomnia Severity Index (ISI) [50] total score is based on a 7-item instrument with high internal consistency (Cronbach alpha 0.9) and high convergent validity for measures of fatigue, quality of life, anxiety, and depression [51]. The total score measures perceived severity of insomnia symptoms. A 0.5 standardized mean difference between treatment and control is considered the clinical significance threshold for studies of chronic insomnia disorder [49].

- Analysis metric: Insomnia Severity Index (4 months post-randomization)
- Method of aggregation: mean
- Time point: 4 months post-randomization

#### Exploratory outcome: nocturia-related quality of life

Specific measurement variable: ICIQ-nocturia quality of life (ICIQ-Nqol) questionnaire [52] total score is measured with a 13-item instrument that assesses psychological distress, sleep, interferences with habits, effect on relationships, limitations on activities and travel, physical energy, concentration, and safety [53].

- Analysis metric: ICIQ-Nqol total score (4 months post-randomization)
- Method of aggregation: mean
- Time point: 4 months post-randomization

#### Exploratory outcome: sleep quality

Specific measurement variable: Pittsburgh Sleep Quality Index (PSQI) [54] total (global) score is one of the most widely used self-report questionnaires for assessing sleep quality (during the past month).

- Analysis metric: PSQI total (global) score value (1 week post-treatment and 4 months post-randomization)
- Method of aggregation: mean
- Time point: 1 week post-treatment and 4 months post-randomization

### Participant timeline {13}

**Fig. 1:**
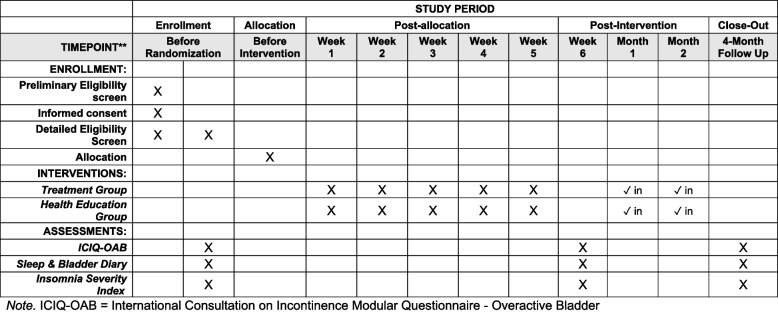
Participant timeline of enrollment, interventions, and assessments

Figure 1 summarizes participant activities over the course of the trial.

### Sample size {14}

Power calculations are informed by parameter assumptions derived from past clinical trials of behavioral interventions for lower urinary tract symptoms and/or sleep disorders conducted by our study team members or consultants, corroborated by trends in our previous integrated nocturia and insomnia intervention feasibility study [36, 37, 55]. We assume an average frequency of at least 16 nocturia episodes per week (~ 2.3 episodes/night) in our baseline trial population, and an average decrease in nocturia frequency of 2 episodes per week (~ 0.3 episodes/night) over 4 months in the control group, given a recent systematic review including 17 trials assessing placebo-response in studies of treatments for nocturia [56]. We also anticipate a clinically meaningful reduction in nocturia frequency (item from the ICIQ-OAB) in the integrated nocturia and insomnia intervention group of at least 7 episodes per week (~ 1 episode per night) over 4 months, leading to a between-group difference in improvement in nocturia frequency of 5 or more episodes per week (0.7 episodes/night) between the integrated nocturia and insomnia intervention and control arms. This effect size represents a meaningful threshold of improvement in nocturia frequency that can be communicated to patients and clinicians, and corresponds to a clinically meaningful improvement of greater than half a standard deviation in the mean nightly nocturia frequency given a range of potential standard deviations [56, 57]. Using an approximation of the covariance matrix of the regression coefficients of the proposed linear mixed effects models (LMMs), we estimate that a sample of 192 participants, randomized in equal proportions to the two arms, will provide > 80% power in 2-sided tests with an experiment-wise type-I error rate of 5% [58], to detect the proposed greater reduction in nocturia frequency of least 5 episodes/week (0.7 episodes/night) associated with integrated intervention versus control intervention, assuming a cumulative loss to follow-up level of less than 15% accumulated at roughly equal rates between sequential follow-up visits. The assumption of loss-to-follow-up < 15% is based on our existing integrated intervention feasibility study, as well as prior trials of behavioral treatment programs for urologic symptoms conducted by the investigators in which early termination was < 15% over similar time periods [36, 37].

### Recruitment {15}

Participants are identified through referrals from clinicians, opt-in/out letters sent to patients who had appointments within the past 18 months or who are scheduled for appointments in the upcoming 18 months at VA GLA or VA Atlanta clinics (e.g., sleep clinic, women’s health, geriatrics, primary care, urology), and local recruitment flyers/posters at our medical centers, UCLA or Emory, and on social media. Patients from these healthcare systems who meet initial screening criteria are invited to an informed consent appointment. Individuals who provide written informed consent (paper or VA-approved e-consent such as DocuSign) are scheduled for an in-depth assessment (which also serves as a baseline assessment for individual who are ultimately randomized) that includes structured interviews with trained research staff, one-week wrist actigraphy, home sleep/bladder diary, home sleep apnea testing for individuals without a prior sleep apnea test and who are high-risk based upon a sleep apnea screening test, blood and urine tests (e.g., hemoglobin A1c, urinalysis) if results are unavailable in medical records, and urinary measures (e.g., residual bladder volume measured with a bladder scan). Each participant’s medical record is also reviewed for exclusion criteria (e.g., bipolar disorder). After initial screening, to minimize exclusions, individuals who are initially excluded for a potentially reversible criterion (e.g., the participant subsequently receives treatment for obstructive sleep apnea, restless legs syndrome, or other medical/mental health conditions) are reconsidered for eligibility.

## Assignment of interventions: allocation

### Sequence generation {16a}

A secure unpredictable allocation sequence (e.g., A B B B A) was generated using randomly permuted blocks, stratified by site and sex, to maximize balance between groups throughout accrual while ensuring the sequence remain unpredictable. The randomization scheme was generated by computer (SAS, SAS Institute Inc., Cary, North Carolina). To avoid manipulation of randomization, a statistical analyst who was not otherwise involved in development of the study database, data cleaning, or data analysis was contracted to program the randomization scheme.

### Concealment mechanism {16b}

Once a candidate is determined to be eligible, arm assignments are made through a controlled (21 CFR part 11 compliant) computer interface (Medrio, Medrio Inc., San Francisco, California), assigning the next randomization number and arm assignment within the strata series. Arm assignment is viewable only by select unblinded staff, and the randomization series is only viewable by the unblinded statistician and coordinating center staff that generate and upload it into the computer database, respectively, accessible only to these unblinded research staff.

### Implementation {16c}

At baseline, after screening data have been reviewed and eligibility has been confirmed by a study coordinator, the coordinator activates the randomization interface, and the next randomization number within the relevant strata is assigned to that participant, with date stamping to ensure the integrity of the randomization assignment order. Once randomized, participants cannot be unrandomized.

## Assignment of interventions: blinding

### Who will be blinded {17a}

While participants are not blinded to treatment group, study staff who conduct assessments are blinded to group assignment, thereby increasing the scientific rigor of our trial. We have also structured the “observable” aspects of the two intervention arms to be similar (e.g., number and frequency of sessions) to facilitate blinding of staff members conducting outcome assessments.

### Procedure for unblinding if needed {17b}

Unblinding will occur if requested by a participant’s clinician who determines knowledge of group assignment is needed to guide care or in the event of a serious adverse event that may be study-related.

## Data collection and management

### Plans for assessment and collection of outcomes {18a}

#### Blinding and assessor training

All primary and secondary outcomes are collected by research staff who are blinded to group assignment. Assessors undergo rigorous training prior to completing assessments, including conducting mock assessments and being observed by experienced research staff prior to conducting the assessments with participants.

As described in the measures section, the ICIQ-OAB is a 4-item instrument that measures nocturia, urgency, frequency, urinary incontinence symptoms, and bother [45, 46]. It is derived from the International Continence Society (ICS) male and Bristol Female Lower Urinary Tract Symptoms questionnaires. It has Grade A validation due to its psychometric testing and published data sets demonstrating validity, reliability, and responsiveness [47]. Sleep diary-derived measures have been compared to polysomnography and actigraphy, with previously reported mean differences between polysomnography and diary of 37.6 min and between actigraphy and diary of 27.8 min in women and mean differences between single-channel electroencephalography and diary of 37.4 min and between actigraphy and diary of 14.7 min in older adults [59, 60]. Insomnia Severity Index has been extensively validated, with Cronbach’s alphas ranging from 0.65 to 0.92 [61]. ISI was assessed in a study comparing cognitive behavioral therapy and pharmacotherapy for late-life insomnia and was found to have concurrent validity with quantitative estimates of sleep measured with sleep diary and polysomnography and to have predictive validity of patient’s perceptions of sleep difficulties, as well as high content validity [62]. The ICIQ-Nqol was assessed for content validity among patients with nocturia and found to have reasonable content validity for women and men [53]. It has Cronbach’s alphas ranging from 0.84 to 0.90 among men with nocturia [53]. The PSQI’s reliability and validity among patients with insomnia is high, with test–retest reliability 0.87 and high correlations between PSQI and sleep diaries, although lower correlations with polysomnography data [63].

### Plans to promote participant retention and complete follow-up {18b}

All study team members are trained to interact with older adults participating in research. Throughout the trial, the research team emphasizes the relevance of the research to other older adults and relevance to the community, including the Veteran community. Gift card incentives are provided for participation in baseline and follow-up assessments. Contact information is updated to ensure that the research team is able to contact the participants for follow-up activities. Participants are encouraged to complete assessments even if they elect not to complete the intervention to which they are assigned.

### Data management {19}

Accurate and complete data collection at each of the study clinics are the responsibility of the site-specific study staff under the supervision of the Site PIs (or other site-specific investigators). Any source documents collected at a study site are reviewed by clinical coordinators at the relevant clinical site under the supervision of the Site PI, who takes initial responsibility for ensuring that they are accurate and complete. Site-specific personnel also have initial responsibility for entering data completely and accurately into the electronic database.

Study data are electronically entered, managed, and edited by clinical coordinators or other study staff at each of the clinical sites using Medrio, a secure, web-based application designed for research data entry and management. The Medrio system automatically generates queries for missing data or out-of-range values based on initial programming by analysts at the UCSF Data Coordinating Center.

The UCSF Data Coordinating Center under the supervision of its Principal Investigator provides a secondary layer of oversight for data accuracy and completeness by tracking data queries posted in the electronic data capture system, prompting site-specific study personnel to address data queries for missing or out-of-range data in a timely manner, and generating study-wide reports for reviews of missing data or data outliers.

Overall data completeness and quality are periodically assessed by the UCSF Data Coordinating Center using measures such as the number of missing data forms and number of outstanding data queries flagged within this system. The PIs review these indicators of data completeness and quality at monthly study-wide meetings involving investigators and personnel at all clinical sites.

Data are managed and edited using Medrio web-based Electronic Data Capture software for Clinical Research, which has been used by members of the investigative team for multiple single- and multicenter trials. Medrio software meets requirements for electronic records and signatures (21 CFR Part 11) as well as Health Insurance Portability and Accountability Act (HIPAA) privacy guidelines. Data entered via Medrio can be accessed from machines on any network, can accommodate multiple simultaneous users, and allows for users to be issued unique password-protected logins. The trial benefits from Medrio’s relational database structure, capacity for internal posting and resolution of data queries, and support for internal tracking of missing visits or data forms. Additional specific features of Medrio that enhance this specific trial include its integrated platforms for administering electronic participant-reported outcome questionnaires, embedded tools for coding adverse events by Medical Dictionary for Regulatory Activities (MedDRA) organ system categories, and tools to allow users to upload study data initially collected offline.

Following procedures developed in our feasibility study, research staff at the VA Atlanta and VA Greater Los Angeles code and enter data into Medrio, and a subset of data will be double entered by staff at the other site and data compared to assess for differences. A standard form is used to record the reason for all exclusions and dropouts, and these data are also entered into Medrio. Data exports are checked with statistical software using automated processes. The data manager reviews accrual, data entry, and eligibility criteria and examines the database for outliers or database entry errors at each site. The Medrio database will then be converted, and data analyses will be completed in SAS 9.4 (SAS Institute, Cary, North Carolina). Medrio does not receive any protected health information or personally identifiable information.

Participant screening, screen failure, and randomization status are provided by the Medrio data manager to National Institutes of Health (NIH) in accordance with NIH reporting requirements.

Data for this study can be entered from any location with secure web authentication, data logging, and Secure Sockets Layer (SSL) encryption. The Medrio system complies with HIPAA regulations. Data are stored on HIPAA-compliant servers, protected by firewalls. The database also incorporates an electronic audit trail to show changes to data after original entry including the date/time and user making the change. Data redundancy is maintained through regular secure off-site data archives.

### Confidentiality {27}

Computer-based records for all research participants (Veteran and non-Veteran) are maintained through the VA Computerized Patient Record System (CPRS), a secure network with password protection, with access provided only to research personnel who are approved by the institutional review board. A unique study identification number is used in lieu of personal identifiers on study records. Consent and privacy authorization forms that contain personal identifiers are stored in locked filing cabinets located in locked offices in approved locations with the research centers. Data will be destroyed according to the Records Control Schedule per VA guidance.

### Plans for collection, laboratory evaluation, and storage of biological specimens for genetic or molecular analysis in this trial/future use {33}

Not applicable. No genetic or molecular analyses are planned during the trial or in the future.

## Statistical methods

### Statistical methods for primary and secondary outcomes {20a}

#### Primary comparative analyses

Our primary analysis will assess the intention-to-treat (ITT) effect of the integrated behavioral therapy program (integrated nocturia and insomnia intervention program) on nocturia frequency compared to the health education group (the primary outcome is single item from ICIQ-OAB 4 months post-randomization). The ITT approach is the standard approach for intervention evaluations in a randomized trial and will include all the randomized participants, including dropouts and non-adherent participants, in the analyses to ensure valid group comparisons. (Generalized) linear mixed effects models (G)LMMs will use all available data from all the randomized participants based on less stringent assumption than completed case analyses. The analyses will adjust for the stratified randomization variables (study site and sex) as suggested in the statistical literature that failure to adjust variables used in the randomization process can result in large *P* values and wide confidence intervals [64]. We expect that the covariates (age, sex, race/ethnicity, education, socioeconomic status, and clinical measures [i.e., presence of nocturnal polyuria or obstructive sleep apnea]) will be balanced between the intervention and control groups at baseline by randomization. However, if by any chance, there are any baseline covariates imbalanced between the intervention and control groups, they will be included in the (G)LMM fixed effects to control for potential confounding.

Aim 1/Hypothesis 1: *Compared to control, participants assigned to the integrated nocturia and insomnia behavioral intervention will demonstrate a greater reduction in nocturia frequency over 4 months*. The primary outcome of nocturia frequency will be measured as the average episodes per night at baseline, 1 week after intervention (approximately 2 months), and 4 months through the ICIQ-OAB questionnaire’s nocturia item. An LMM will be used to evaluate the group difference over time in average nocturia frequency per night, with group (the integrated intervention vs. control), time, time*group interaction, and stratified randomization variables (study site and sex) in fixed effects, and nested two-level random effects to account for correlations due to clustering by study site and participants. The time*group interaction will provide the estimated ITT effect of the integrated intervention on nocturia frequency over time. Imbalanced baseline variables, if any will be included in the LMM.

#### Secondary comparative analyses

Aim 2/Hypothesis 2: *Compared to control, participants assigned to integrated nocturia and insomnia behavioral intervention will have greater reduction in self-reported wake after sleep onset and Insomnia Severity Index over the 4-month follow-up period*. Secondary outcomes, patient-reported wake after sleep onset and ISI, will be modeled similarly with a (G)LMM in the original scale or transformed scale as appropriate, where group, time, time*group interaction, and stratified randomization variables (study site and sex) will be included in fixed effects, and random effects will be included for nested clustering by site and participant to account for correlations due to clustering. If the distributions of the secondary outcomes are skewed and no good transformation is found, the outcomes will be categorized into categories based on quantiles or clinically meaningful values and then modeled with GLMMs using cumulative logit or generalized logit link for multinomial data. Similar fixed and random effects will be included in GLMMs. Due to the exploratory feature, no adjustment will be made for the multiple comparisons, but results will be presented as secondary outcome analyses. Secondary analyses similar to those proposed in Aim 1 will be performed on the secondary outcomes of patient-reported wake after sleep onset and ISI.

### Interim analyses {21b}

The behavioral interventions are considered to be minimal risk, and the data collection procedures impose only modest burden on participants. Accordingly, no interim analyses are planned, as there is not a compelling reason or rationale for early termination of the trial, even if any differences in the outcomes begin to emerge earlier than expected, and analyses involving preliminary data could lead to spurious conclusions that may impair the credibility of the findings. However, if the Data and Safety Monitor/Medical Safety Officer (DSM/MSO) requests interim outcomes analyses based on new evidence from the trial or other researchers such that the risks of study treatments are substantially greater than anticipated, the interim analyses will be performed. Similarly, the DSM/MSO may terminate the trial if trial safety measure data suggest safety issues or if recruitment and retention or data quality are so impaired that the study aims could not be met.

### Methods for additional analyses (e.g., subgroup analyses) {20b}

ICIQ-Nqol scores and PSQI Global scores will be modeled similarly with (G)LMMs in their original scale or transformed scale as appropriate, with similar fixed effects and random effects as proposed in Aims 1 and 2. If the distributions of the ICIQ-Nqol and PSQI Global scores are skewed and no good transformation is found, the outcomes will be categorized into categories based on quantiles or clinically meaningful values and then modeled with GLMMs using cumulative logit or generalized logit link for multinomial data. Similar fixed and random effects will be included in GLMMs. Due to the exploratory feature, no adjustment will be made for the multiple comparisons, but results will be presented as secondary outcome analyses. Similar secondary analyses as those proposed in Aim 1 will be performed on the secondary outcomes ICIQ-Nqol and PSQI.

### Methods in analysis to handle protocol non-adherence and any statistical methods to handle missing data {20c}

Participant drop-out or loss-to-follow-up levels are expected to be less than 15%, based on observed participant drop-out levels from our multi-center feasibility study. (G)LMM will use all available data in the analyses without dropping participants or including only “completers”, and its estimates are consistent under the assumption of missing at random conditional on observables, a less stringent assumption than the missing completely at random in complete case analysis. The assumption of non-informative participant drop-out will be assessed by comparing baseline characteristics and early post-randomization outcomes of trial dropouts vs. non-dropouts. If participant drop-out rates differ between groups, sensitivity analyses to evaluate the effects of drop-out on estimates of treatment effects will be conducted. Sensitivity analysis will be conducted to assess how much the results from the primary analysis will change 1) if the (G)LMM includes baseline covariates different between dropout and completed participants and 2) if multiple imputation and/or pattern mixture model under plausible informative missingness assumptions to overcome missing data related to participant drop-out is considered. Potential outcome-based causal inference approaches will be used to evaluate the treatment effects while accounting for participants’ nonadherence [65, 66].

### Plans to give access to the full protocol, participant-level data, and statistical code {31c}

The project will result in a deidentified dataset that will contain demographic, subjective and objective (edited and averaged actigraphy data) sleep, and other health-related data of enrolled participants. Materials generated under the project will be disseminated in accordance with the policies at the data coordinating center, and NIH. Data products will be made available without cost to researchers, students, or analysts. To our knowledge, no common data standards exist for multiple key variables examined in this trial, but the deidentified data set will be available in a common format (.csv). User registration and a data use agreement will be required in order to access or download files. As part of the registration process, users must agree to the conditions of use governing access to the public release data, including restrictions against attempting to identify study participants, destruction of the data after analyses are completed, reporting responsibilities, restrictions on redistribution of data to third parties, and proper acknowledgement of the data source. Registered users will receive the data dictionary and user support, as well as information related to errors in the data, future release, workshops, and publication lists. The information provided to users will not be used for commercial purposes and will not be redistributed to third parties. Registered users will be provided access to the full protocol. Statistical code will be made available upon request.

## Oversight and monitoring

### Composition of the coordinating center and trial steering committee {5d}

#### Coordinating center

UCSF is serving as the data coordinating center for this trial, under the supervision of Alison Huang, MD, MAS, MPhil, an internal medicine physician and clinical trialist experienced with conducting research on urinary symptoms in older adults. Our steering committee is composed of the MPIs (Camille Vaughan, MD, MS and Constance Fung, MD, MSHS) and key co-Investigators (Alison Huang, MD, MAS, MPhil and Alayne Markland, DO, MSc).

### Composition of the data monitoring committee, its role and reporting structure {21a}

The safety of participants and conduct of the study are further monitored by a DSM/MSO, appointed by and acting in an advisory capacity to the National Institute on Aging (NIA) and the investigator. The DSM/MSO is charged with periodically reviewing the conduct and outcomes of the study and providing feedback to the investigators and the NIA, with particular attention to protecting the safety of the participants. The DSM/MSO is independent of the institution and investigators participating in the study and has no financial, scientific, or other conflict of interest with the trial. The DSM/MSO meets approximately twice yearly during the data collection phase of the trial, unless the NIA Program Official or DSM/MSO requests more frequent meetings.

### Adverse event reporting and harms {22}

New adverse events (AEs), serious adverse events (SAEs), or unanticipated problems (UPs) will be considered reportable any time after the Baseline Visit when participants are randomized until the 4-month follow-up. At each study visit, participants are asked if they have had any significant change in health status since the prior study visit, and if so, a follow-up question about the nature of the change will be asked. Medical or psychiatric conditions that are present at the time that participants are screened are considered to reflect participants’ baseline status and will not be reported as adverse events. However, if a participant’s condition deteriorates at any time during the study, this deterioration will be recorded as an AE even if it is related to a pre-existing condition.

Adverse events are characterized by clinical coordinators at each clinical site in collaboration with the Site PI or other investigators if appropriate. The clinical coordinator in conjunction with the Site PI documents events on standardized AE or SAE forms. The Site PI reviews information about individual AEs collected by coordinators at least twice monthly to guide determinations about the relationship to study interventions, expectedness, and severity. In cases where an AE may be a SAE or represent an Unanticipated Problem, the coordinator will more urgently bring the event to the attention of the Site PI.

The trial follows the VA reporting requirements and timelines for reporting, including ensuring verbal notification of the IRB immediately upon becoming aware of any local research death that is both unanticipated and related to the research, written notification of the IRB within 5 business days after becoming aware of any local SAE that is both unanticipated and related to the research, written notification of the IRB within 5 business days after becoming aware of any serious problem that is both unanticipated and related to the research. SAEs will be reported to the DSM/MSO, and the NIA within time frames specified by these entities, depending on whether events (a) are possibly related to study participation, (b) are unexpected, and/or (c) result in death or are life-threatening:

Any SAEs that are unexpected but potentially related to the intervention will be reported to the DSM/MSO and the NIA within 48 h of the Site PI’s awareness of the event. The team will report other SAEs to NIA Program Officer and to the DSM/MSO in the semi-annual AE summary provided to the DSM/MSO and the NIA, unless otherwise requested. Non-serious AEs that are considered to be UPs, by virtue of being unexpected but possibly related to study participation, will be reported to the DSM/MSO and the NIA within 5 working days of the Principal Investigators’ awareness of the event. The team will report non-serious AEs that are not considered to be UPs to the DSM/MSO and the NIA in the semi-annual AE summary provided to the DSM/MSO and the NIA.

### Frequency and plans for auditing trial conduct {23}

The VA Greater Los Angeles conducts a monthly audit of informed consent process completion. Atlanta VA conducts an annual audit of informed consent process completion. The NIA monitors enrollment through its Clinical Research Operations & Management System (CROMS). There are no current plans for external audits of trial conduct.

### Plans for communicating important protocol amendments to relevant parties (e.g., trial participants, ethical committees) {25}

Important protocol modifications are approved by local IRBs and DSM/MSO prior to implementation and will be reported in trial registries. The MPIs will discuss with the NIA Program Official any protocol modifications that would impact the ability of the trial to achieve the specific aims of the funded study. Any changes that would impact the risk/benefit of enrolled participants will be communicated to participants.

### Dissemination plans {31a}

The study team plans to disseminate findings through national scientific and professional conferences such as the American Geriatrics Society, American Academy of Sleep Medicine, Sleep Research Society, and American Urological Association conferences, peer-reviewed journal publications, and the VA Geriatric Research, Education and Clinical Center network. Results may be communicated to study participants through an IRB-approved study newsletter. Main results will be posted in clinicaltrials.gov no later than 1 year after the primary completion date.

## Discussion

This multi-site trial is designed to provide rigorous evidence to evaluate the impact of the integrated behavioral treatment for older adults with overlapping nocturia and insomnia symptoms. It will yield an integrated bladder-sleep diary that can be used to assess patients with nocturia and insomnia symptoms. In addition, the intervention will be delivered by staff from different clinical backgrounds (clinical psychologists, nurse practitioners, physician assistants) to inform future implementation efforts.

With the enrollment sites based at two VA medical centers (one in Los Angeles and one in Atlanta), recruitment sites inclusive of VA and academic medical centers, and the data coordinating center based at an academic UCSF site, this project demonstrates extensive collaboration among federal, state, and private entities to tackle nocturia, a prevalent but undertreated condition that impacts the quality of life of many older adults. The funding (grant from NIH/NIA and in-kind support from the VA Geriatric Research, Education and Clinical Centers) also reflects collaborative effort from different federal agencies. The elaborate and extensive protocol that enables multiple organizations to participate in the multi-site trial was made possible by the NIA planning grant [67]. Furthermore, the collaboration among the sites provides the opportunity to expand recruitment efforts (e.g., to academic medical centers) if the teams experience challenges in recruitment overall or in recruiting women at the VA medical centers. The NIA’s CROMS system provides close oversight of the recruitment process.

Collaboration among the two VA Geriatric Research, Education and Clinical Centers that has enabled sharing of resources and more efficient recruitment (e.g., data analyst conducting data extractions for both Los Angeles and Atlanta). The option to deliver the interventions through video encounters using the VA’s video visits and the collaboration among the VA medical centers across the federal health system is also enabling interventionists at either site to conduct the intervention visits, which provides more flexible appointment times for participants during intervention.

Future plans include a multi-center implementation trial that is informed by patients, caregivers, and other stakeholders to assess the clinical effectiveness of the integrated program among patients recruited from a variety of healthcare systems.

### Trial status

Recruitment began in December 2023 and is expected to be completed in June 2028.

The protocol version reported in this paper is version March 10, 2025 (v6).

## Data Availability

The project will result in a deidentified dataset that will contain demographic, subjective and objective (edited and averaged actigraphy data) sleep, and other health-related data of enrolled participants. Deidentification will take place at the time of data entry to the data coordinating center. Materials generated under the project will be disseminated in accordance with the policies at UCSF, which is the data coordinating center for the study, and NIH. User registration and a data use agreement will be required to access or download files. As part of the registration process, users must agree to the conditions of use governing access to the public release data, including restrictions against attempting to identify study participants, destruction of the data after analyses are completed, reporting responsibilities, restrictions on redistribution of data to third parties, and proper acknowledgement of the data source. The information provided to users must not be used for commercial purposes and will not be redistributed to third parties.

## Abbreviations

AE: Adverse events
CBT-I: Cognitive behavioral therapy for insomnia
CFR: Code of Federal Regulations
CPRS: Computerized Patient Record System
CROMS: Clinical Research Operations & Management System
DO: Doctor of Osteopathic medicine
DSM: Data and Safety Monitor
GLA: Greater Los Angeles
(G)LMM: Generalized linear mixed effects models
HIPAA: Health Insurance Portability and Accountability Act
ICIQ-Nqol: International Consultation on Incontinence Questionnaire-nocturia quality of life
ICIQ-OAB: International Consultation on Incontinence Questionnaire-Overactive bladder
ICS: International Continence Society
ITT: Intention-to-treat
IRB: Institutional review board
ISI: Insomnia Severity Index
LMM: Linear mixed effects models
MD: Doctor of Medicine
MedDRA: Medical Dictionary for Regulatory Activities
MINT: Multi-site randomized trial testing an integrated behavioral treatment for improving nocturia and insomnia symptoms in older adults
MPI: Multiple Principal Investigator
MSO: Medical Safety Officer
NIA: National Institute on Aging
NIH: National Institutes of Health
PI: Principal investigator
PSQI: Pittsburgh Sleep Quality Index
SAE: Serious adverse events
SSL: Secure Sockets Layer
UCLA: University of California, Los Angeles
UCSF: University of California San Francisco
UP: Unanticipated problems
VA: Department of Veterans Affairs

## References

[1] Markland AD, Vaughan CP, Johnson TM, Goode PS, Redden DT, Burgio KL. Prevalence of nocturia in United States men: results from the National Health and Nutrition Examination Survey. J Urol. 2011;185(3):998–1002. 21251675 10.1016/j.juro.2010.10.083

[2] Lowenstein L, Brubaker L, Kenton K, Kramer H, Shott S, FitzGerald MP. Prevalence and impact of nocturia in a urogynecologic population. Int Urogynecol J Pelvic Floor Dysfunct. 2007;18(9):1049–52. 17216553 10.1007/s00192-006-0289-1

[3] Tikkinen KAO, Tammela TLJ, Huhtala H, Auvinen A. Is nocturia equally common among men and women? A population based study in Finland. J Urol. 2006;175(2):596–600. 16407003 10.1016/S0022-5347(05)00245-4

[4] Coyne KS, Zhou Z, Bhattacharyya SK, Thompson CL, Dhawan R, Versi E. The prevalence of nocturia and its effect on health-related quality of life and sleep in a community sample in the USA. BJU Int. 2003;92(9):948–54. 14632853 10.1111/j.1464-410x.2003.04527.x

[5] Vaughan CP, Fung CH, Huang AJ, Johnson 2nd TM, Markland AD. Differences in the association of nocturia and functional outcomes of sleep by age and gender: a cross-sectional, population-based study. Clin Ther. 2016;38(11):2386-93.e1. 10.1016/j.clinthera.2016.09.009 PMC5110388 27751673

[6] DuBeau CE, Yalla SV, Resnick NM. Implications of the most bothersome prostatism symptom for clinical care and outcomes research. J Am Geriatr Soc. 1995;43(9):985–92. 7544816 10.1111/j.1532-5415.1995.tb05562.x

[7] Agarwal A, Eryuzlu LN, Cartwright R, Thorlund K, Tammela TLJ, Guyatt GH, et al. What is the most bothersome lower urinary tract symptom? Individual- and population-level perspectives for both men and women. Eur Urol. 2014;65(6):1211–7. 24486308 10.1016/j.eururo.2014.01.019 PMC4018666

[8] Tikkinen KAO, Johnson II TM, Tammela TLJ, Sintonen H, Haukka J, Huhtala H, et al. Nocturia frequency, bother, and quality of life: how often is too often? A population-based study in Finland. Eur Urol. 2010;57(3):488–98. 10.1016/j.eururo.2009.03.080 19361907

[9] Bliwise DL, Rosen RC, Baum N. Impact of nocturia on sleep and quality of life: a brief, selected review for the International Consultation on Incontinence Research Society (ICI-RS) nocturia think tank. Neurourol Urodyn. 2014;33(S1):S15-8. 24729148 10.1002/nau.22585

[10] Yu HJ, Chen FY, Huang P-C, Chen THH, Chie WC, Liu CY. Impact of nocturia on symptom-specific quality of life among community-dwelling adults aged 40 years and older. Urology. 2006;67(4):713–8. 16566966 10.1016/j.urology.2005.10.054

[11] Vaughan CP, Brown CJ, Goode PS, Burgio KL, Allman RM, Johnson TM. The association of nocturia with incident falls in an elderly community-dwelling cohort. Int J Clin Pract. 2010;64(5):577–83. 20456212 10.1111/j.1742-1241.2009.02326.x PMC3222329

[12] Parthasarathy S, Fitzgerald M, Goodwin JL, Unruh M, Guerra S, Quan SF. Nocturia, sleep-disordered breathing, and cardiovascular morbidity in a community-based cohort. PLoS One. 2012;7(2):e30969. 22328924 10.1371/journal.pone.0030969 PMC3273490

[13] Bliwise DL, Foley DJ, Vitiello MV, Ansari FP, Ancoli-Israel S, Walsh JK. Nocturia and disturbed sleep in the elderly. Sleep Med. 2009;10(5):540–8. 18703381 10.1016/j.sleep.2008.04.002 PMC2735085

[14] Kupelian V, Fitzgerald MP, Kaplan SA, Norgaard JP, Chiu GR, Rosen RC. Association of nocturia and mortality: results from the third National Health and Nutrition Examination Survey. J Urol. 2011;185(2):571–7. 21168875 10.1016/j.juro.2010.09.108

[15] Endeshaw YW, Schwartz AV, Stone KL, Caserotti P, Harris T, Smagula S, et al. Nocturia, insomnia symptoms, and mortality among older men: the health, aging and body composition study. J Clin Sleep Med. 2016;12:789–96. 26951411 10.5664/jcsm.5870 PMC4877310

[16] Johnson TV, Abbasi A, Ehrlich SS, Kleris RS, Raison CL, Master VA. Nocturia associated with depressive symptoms. Urology. 2011;77(1):183–6. 20599253 10.1016/j.urology.2010.04.048

[17] Bliwise DL, Wagg A, Sand PK. Nocturia: a highly prevalent disorder with multifaceted consequences. Urology. 2019;133S:3–13. 31310770 10.1016/j.urology.2019.07.005

[18] Tinetti ME, Williams CS, Gill TM. Dizziness among older adults: a possible geriatric syndrome. Ann Intern Med. 2000;132(5):337–44. 10691583 10.7326/0003-4819-132-5-200003070-00002

[19] Hirayama A, Torimoto K, Mastusita C, Okamoto N, Morikawa M, Tanaka N, et al. Evaluation of factors influencing the natural history of nocturia in elderly subjects: results of the Fujiwara-kyo study. J Urol. 2013;189(3):980–6. 23036982 10.1016/j.juro.2012.09.118

[20] Tikkinen KAO, Auvinen A, Johnson TM II, Weiss JP, Keranen T, Tiitinen A, et al. A systematic evaluation of factors associated with nocturia–The population-based FINNO study. Am J Epidemiol. 2009;170(3):361–8. 19515794 10.1093/aje/kwp133 PMC2714949

[21] Wennberg AL, Altman D, Lundholm C, Klint A, Iliadou A, Peeker R, et al. Genetic influences are important for most but not all lower urinary tract symptoms: a population-based survey in a cohort of adult Swedish twins. Eur Urol. 2011;59(6):1032–8. 21420232 10.1016/j.eururo.2011.03.007 PMC3101479

[22] Weiss JP, Juul KV, Wein A. Management of nocturia: the role of antidiuretic pharmacotherapy. Neurourol Urodyn. 2014;33:S19-24. 24729149 10.1002/nau.22592

[23] Smith M, Dawson S, Andrews RC, Eriksson SH, Selsick H, Skyrme-Jones A, et al. Evaluation and treatment in urology for nocturia caused by nonurological mechanisms: guidance from the PLANET study. Eur Urol Focus. 2022;8(1):89–97. 35101453 10.1016/j.euf.2022.01.007

[24] Vaughan CP, Eisenstein R, Bliwise DL, Endeshaw YK, Nagamia ZJ, Wolf RA, et al. Self-rated sleep characteristics and bother from nocturia. Int J Clin Pract. 2012;66(4):369–73. 22356249 10.1111/j.1742-1241.2011.02868.x

[25] Bliwise DL, Foley DJ, Vitiello MV, Ansari FP, Ancoli-Israel S, Walsh JK. Nocturia and disturbed sleep in the elderly. Sleep Med. 2009;10(5):540–8. 18703381 10.1016/j.sleep.2008.04.002 PMC2735085

[26] Zeitzer JM, Bliwise DL, Hernandez B, Friedman L, Yesavage JA. Nocturia compounds nocturnal wakefulness in older individuals with insomnia. J Clin Sleep Med. 2013;9(3):259–62. 23493881 10.5664/jcsm.2492 PMC3578689

[27] Sakalis VI, Karavitakis M, Bedretdinova D, Bach T, Bosch JLHR, Gacci M, et al. Medical treatment of nocturia in men with lower urinary tract symptoms: systematic review by the European association of urology guidelines panel for male lower urinary tract symptoms. Eur Urol. 2017;72(5):757–69. 28666669 10.1016/j.eururo.2017.06.010

[28] Bedretdinova D, Ambühl D, Omar MI, Sakalis V, Thiruchelvam N, Schneider M, et al. What is the most effective treatment for nocturia or nocturnal incontinence in adult women? Eur Urol Focus. 2021;7(2):453–63. 32061540 10.1016/j.euf.2020.01.012

[29] Fralick M, Schneeweiss S, Wallis CJD, Jung EH, Kesselheim AS. Desmopressin and the risk of hyponatremia: a population-based cohort study. PLoS Med. 2019;16(10):e1002930. 31634354 10.1371/journal.pmed.1002930 PMC6802819

[30] Suvada K, Plantinga L, Vaughan CP, Markland AD, Mirk A, Burgio KL, et al. Comorbidities, age, and polypharmacy limit the use by US older adults with nocturia of the only FDA-approved drugs for the symptom. Clin Ther. 2020;42(12):e259–74. 33257091 10.1016/j.clinthera.2020.11.003 PMC8811749

[31] Desmopressin acetate: 2023; Available from: https://dailymed.nlm.nih.gov/dailymed/fda/fdaDrugXsl.cfm?setid=539d7da2-72b8-45f9-836c-4e3351dd488e&type=display#:~:text=FULL%20PRESCRIBING%20INFORMATION-,WARNING:%20HYPONATREMIA,Warnings%20and%20Precautions%20(5.1)%5D. Accessed 6 Aug 2025.

[32] Simaioforidis V, Papatsoris AG, Chrisofos M, Chrisafis M, Koritsiadis S, Deliveliotis C. Tamsulosin versus transurethral resection of the prostate: effect on nocturia as a result of benign prostatic hyperplasia. Int J Urol. 2011;18(3):243–8. 21332817 10.1111/j.1442-2042.2010.02704.x

[33] Marshall SD, Raskolnikov D, Blanker MH, Hashim H, Kupelian V, Tikkinen KAO, et al. Nocturia: current levels of evidence and recommendations from the international consultation on male lower urinary tract symptoms. Urology. 2015;85(6):1291–9. 25881866 10.1016/j.urology.2015.02.043

[34] Gormley EA, Lightner DJ, Burgio KL, Chai TC, Clemens JQ, Culkin DJ, et al. Diagnosis and treatment of overactive bladder (Non-Neurogenic) in adults: AUA/SUFU guideline. J Urol. 2012;188(1):2455–63. 23098785 10.1016/j.juro.2012.09.079

[35] Gormley EA, Lightner DJ, Faraday M, Vasavada SP. Diagnosis and treatment of overactive bladder (non-neurogenic) in adults: AUA/SUFU guideline amendment. J Urol. 2015;193(5):1572–80. 25623739 10.1016/j.juro.2015.01.087

[36] Vaughan CP, Endeshaw Y, Nagamia Z, Ouslander JG, Johnson TM. A multicomponent behavioral and drug intervention for nocturia in elderly men: rationale and pilot results. BJU Int. 2009;104(1):69–74. 19220247 10.1111/j.1464-410X.2009.08353.x

[37] Johnson TM II, Vaughan CP, Goode PS, Bliwise DL, Markland AD, Huisingh C, et al. Pilot results from a randomized trial in men comparing alpha-adrenergic antagonist versus behavior and exercise for nocturia and sleep. Clin Ther. 2016;38(11):2394-406.e3. 10.1016/j.clinthera.2016.10.001 28029383

[38] Vaughan CP, Markland AD, Huang AJ, Alessi CA, Guzman A, Martin JL, et al. Considerations for integrated cognitive behavioral treatment for older adults with coexisting nocturia and insomnia. Age Ageing. 2022;51(2):afac024. 35165690 10.1093/ageing/afac024 PMC8844541

[39] Edinger JD, Carney C. Overcoming insomnia: a cognitive-behavioral therapy approach, workbook. 2nd ed. Oxford University Press; 2014.

[40] Sandhu JS, Bixler BR, Dahm P, Goueli R, Kirkby E, Stoffel JT, et al. Management of lower urinary tract symptoms attributed to benign prostatic hyperplasia (BPH): AUA guideline amendment 2023. J Urol. 2024;211(1):11–9. 37706750 10.1097/JU.0000000000003698

[41] Fung CH, Vaughan CP, Markland AD, Huang AJ, Mitchell MN, Bliwise DL, Ancoli-Israel S, Redline S, Alessi CA, Stone K. Nocturia is associated with poor sleep quality among older women in the Study of Osteoporotic Fractures. J Am Geriatri Soc. 2017;65(S1):S134. 10.1111/jgs.15027 PMC5681421 28914959

[42] Tyagi S, Resnick NM, Perera S, Monk TH, Hall MH, Buysse DJ. Behavioral treatment of insomnia: also effective for nocturia. J Am Geriatr Soc. 2014;62(1):54–60. 24383406 10.1111/jgs.12609 PMC4055528

[43] Araujo AB, Yaggi HK, Yang M, McVary KT, Fang SC, Bliwise DL. Sleep-related problems and urologic symptoms: testing the hypothesis of bi-directionality in a longitudinal, population-based study. J Urol. 2014;191(1):100–6. 23867307 10.1016/j.juro.2013.07.011 PMC3865224

[44] Burgio KL, Goode PS, Johnson TM, Hammontree L, Ouslander JG, Markland AD, et al. Behavioral versus drug treatment for overactive bladder in men: the male overactive bladder treatment in veterans (MOTIVE) Trial. J Am Geriatr Soc. 2011;59(12):2209–16. 22092152 10.1111/j.1532-5415.2011.03724.x

[45] Jackson S, Donovan J, Brookes S, Eckford S, Swithinbank L, Abrams P. The Bristol female lower urinary tract symptoms questionnaire: development and psychometric testing. Br J Urol. 1996;77(6):805–12. 8705212 10.1046/j.1464-410x.1996.00186.x

[46] Donovan JL, Abrams P, Peters TJ, Kay HE, Reynard J, Chapple C, et al. The ICS–‘BPH’ study: the psychometric validity and reliability of the ICSmale questionnaire. Br J Urol. 1996;77(4):554–62. 8777617 10.1046/j.1464-410x.1996.93013.x

[47] Shy M, Fletcher SG. Objective evaluation of overactive bladder: which surveys should I use? Curr Bladder Dysfunct Rep. 2013;8(1):45–50. 23439804 10.1007/s11884-012-0167-2 PMC3579666

[48] Carney CE, Buysse DJ, Ancoli-Israel S, Edinger JD, Krystal AD, Lichstein KL, et al. The consensus sleep diary: standardizing prospective sleep self-monitoring. Sleep. 2012;35(2):287–302. 22294820 10.5665/sleep.1642 PMC3250369

[49] Edinger JD, Arnedt JT, Bertisch SM, Carney CE, Harrington JJ, Lichstein KL, et al. Behavioral and psychological treatments for chronic insomnia disorder in adults: an American Academy of Sleep Medicine systematic review, meta-analysis, and GRADE assessment. J Clin Sleep Med. 2021;17(2):263–98. 33164741 10.5664/jcsm.8988 PMC7853211

[50] Bastien CH, Vallières A, Morin CM. Validation of the insomnia severity index as an outcome measure for insomnia research. Sleep Med. 2001;2(4):297–307. 11438246 10.1016/s1389-9457(00)00065-4

[51] Morin CM, Belleville G, Belanger L, Ivers H. The insomnia severity index: psychometric indicators to detect insomnia cases and evaluate treatment response. Sleep. 2011;34(5):601–8. 21532953 10.1093/sleep/34.5.601 PMC3079939

[52] Kelleher CJ, Cardozo LD, Khullar V, Salvatore SC. A new questionnaire to assess the quality of life of urinary incontinent women. BJOG: An International Journal of Obstetrics & Gynaecology. 1997;104(12):1374–9. 10.1111/j.1471-0528.1997.tb11006.x 9422015

[53] Mock LL, Parmelee PA, Kutner N, Scott J, Johnson TM, 2nd. Content validation of symptom-specific nocturia quality-of-life instrument developed in men: issues expressed by women, as well as men. Urology. 2008;72(4):736-42. 10.1016/j.urology.2008.04.014 18684494

[54] Buysse DJ, Reynolds ICF, Monk TH, Berman SR, Kupfer DJ. The Pittsburgh sleep quality index: a new instrument for psychiatric practice and research. Psychiatr Res. 1989;28(2):193–213. 10.1016/0165-1781(89)90047-4 2748771

[55] Fung C, Alessi C, Song Y, Vaughan CP, Markland AD, Huang A, et al., editors. Behavioral therapies for insomnia improve nocturia in older women Veterans. 21st International Association of Gerontology and Geriatrics (IAGG) World Congress of Gerontology and Geriatrics; 2017; San Francisco.

[56] Mostafaei H, Janisch F, Mori K, Quhal F, Pradere B, Hajebrahimi S, et al. Placebo response in patients with oral therapy for overactive bladder: a systematic review and meta-analysis. Eur Urol Focus. 2022;8(1):239–52. 33674256 10.1016/j.euf.2021.02.005

[57] Norman GR, Sloan JA, Wyrwich KW. Interpretation of changes in health-related quality of life: the remarkable universality of half a standard deviation. Med Care. 2003;41(5):582–92. 12719681 10.1097/01.MLR.0000062554.74615.4C

[58] Seghezzo G, Van Hoecke Y, James L, Davoren D, Williamson E, Pearce N, et al. Feasibility study of assessing the Preclinical Alzheimer Cognitive Composite (PACC) score via videoconferencing. J Neurol. 2021;268(6):2228–37. 33496862 10.1007/s00415-021-10403-1 PMC7836343

[59] Lehrer HM, Yao Z, Krafty RT, Evans MA, Buysse DJ, Kravitz HM, et al. Comparing polysomnography, actigraphy, and sleep diary in the home environment: the study of women’s health across the nation (SWAN) sleep study. SLEEP Adv. 2022;3(1):zpac001. 35296109 10.1093/sleepadvances/zpac001 PMC8918428

[60] Chou CA, Toedebusch CD, Redrick T, Freund D, McLeland JS, Morris JC, et al. Comparison of single-channel EEG, actigraphy, and sleep diary in cognitively normal and mildly impaired older adults. Sleep Adv. 2020;1(1):zpaa006. 33644758 10.1093/sleepadvances/zpaa006 PMC7898727

[61] Cerri LQ, Justo MC, Clemente V, Gomes AA, Pereira AS, Marques DR. Insomnia severity index: a reliability generalisation meta-analysis. J Sleep Res. 2023;32(4):e13835. 36737257 10.1111/jsr.13835

[62] Bastien CH, Vallieres A, Morin CM. Validation of the insomnia severity index as an outcome measure for insomnia research. Sleep Med. 2001;2(4):297–307. 11438246 10.1016/s1389-9457(00)00065-4

[63] Backhaus J, Junghanns K, Broocks A, Riemann D, Hohagen F. Test-retest reliability and validity of the Pittsburgh sleep quality index in primary insomnia. J Psychosom Res. 2002;53(3):737–40. 12217446 10.1016/s0022-3999(02)00330-6

[64] Kahan BC, Morris TP. Reporting and analysis of trials using stratified randomisation in leading medical journals: review and reanalysis. BMJ. 2012;345:e5840. 22983531 10.1136/bmj.e5840 PMC3444136

[65] Angrist JD, Imbens GW, Rubin DB. Identification of causal effects using instrumental variables. J Am Stat Assoc. 1996;91(434):444–55.

[66] Cheng J, Qin J, Zhang B. Semiparametric estimation and inference for distributional and general treatment effects. J R Stat Soc Ser B Stat Methodol. 2009;71(4):881–904.

[67] Fung CH, Huang AJ, Markland AD, Schembri M, Martin JL, Bliwise DL, et al. A multisite feasibility study of integrated cognitive-behavioral treatment for co-existing nocturia and chronic insomnia. J Am Geriatr Soc. 2025;73(2):558–65. 39389800 10.1111/jgs.19214 PMC11825994

